# Methotrexate for refractory adult atopic dermatitis leads to alterations in cutaneous IL-31 and IL-31RA expression^⋆^

**DOI:** 10.1016/j.abd.2023.01.002

**Published:** 2023-09-18

**Authors:** Luciana Paula Samorano, Kelly Cristina Gomes Manfrere, Naiura Vieira Pereira, Roberto Takaoka, Neusa Yuriko Sakai Valente, Mirian Nacagami Sotto, Luiz Fernando Ferraz Silva, Maria Notomi Sato, Valeria Aoki

**Affiliations:** a Department of Dermatology, Faculty of Medicine, Universidade de São Paulo, São Paulo, SP, Brazil; b Department of Dermatology, Laboratório de Dermatologia e Imunodeficiências (LIM-56), Faculty of Medicine, Universidade de São Paulo, São Paulo, SP, Brazil; c Department of Pathology, Faculty of Medicine, Universidade de São Paulo, São Paulo, SP, Brazil

**Keywords:** Dermatitis, atopic, Inflammation, Interleukins, Methotrexate, Pruritus, Receptors, interleukins

## Abstract

**Background:**

Methotrexate (MTX) is an alternative treatment for patients with moderate/severe atopic dermatitis (AD).

**Objective:**

The authors evaluated the effect of MTX on the cutaneous expression of cytokines and chemokines that are involved in the inflammatory response in adult AD patients who received treatment with methotrexate for 24 weeks.

**Methods:**

The authors conducted a prospective single-institution cohort study with 12 adults with moderate/severe AD who received oral MTX (15 mg/wk for 24 wks) and 10 non-atopic matched controls. The comparison was made of skin biopsies of lesional and non-lesional skin, pre- and post MTX treatment. The authors analyzed mean epidermal thickness and expression of IL-31, IL-31RA, OSMR, TSLP, Ki67, IL-4 mRNA, IL-6, IL-10, TNF-α, IFN-γ, TARC, and CCL-22.

**Results:**

There was a reduction in mean epidermal thickness (p = 0.021), an increase in IL-31RA expression (immunohistochemistry) in the epidermis (p = 0.016) and a decrease in IL-31 gene expression (p = 0.019) on lesional AD skin post-MTX treatment. No significant changes in the cutaneous expression of the other evaluated markers were identified.

**Study limitations:**

Small sample size and limited length of follow-up.

**Conclusions:**

Treatment with MTX in adults with moderate/severe AD reduced epidermal hyperplasia and changed the cutaneous expression of inflammatory cytokines and receptors that are mainly related to pruritus, including IL-31 and IL-31RA.

**Trial registration:**

ClinicalTrials.gov Identifier: NCT03327116.

## Introduction

Atopic Dermatitis (AD) is a chronic and pruriginous skin disease with a complex pathophysiology. Several factors are involved, including fundamental skin barrier defects and immune dysfunction that results in inflammation, as well as the typical eczematous lesions.^1^, ^2^, ^3^

An increase in Th2 (IL-4, IL-10, IL-13) and Th22 (IL-22) cytokines and chemokines^4^, ^5^ is seen in the acute phase of AD. Moreover, an increase in the expression of Th1 (IL-12, IFN-γ) and Th17 (IL-17) cytokines occurs in the chronic phase. These observations are mainly seen in studies that focus on children^6^ and the Asian population.^7^

Other cytokines have been identified in AD inflammation. These include TSLP (thymic stromal lymphopoietin) and IL-31. Epidermal keratinocytes express TSLP, thereby promoting the activation of myeloid dendritic cells, as well as Th2 and Th22 cells. As a result, there is a promotion of pruritus.^8^, ^9^ IL-31 expression promotes Th2 cell-mediated inflammation. There may also here been an association with pruritus.^10^ Signaling occurs through a heterodimeric receptor complex of Oncostatin M Receptor (OSMR) and IL-31 alpha-receptor (IL-31RA) subunits.^11^

AD treatment should focus on controlling inflammation and repairing skin barrier changes. As a result, Methotrexate (MTX) may serve as a treatment for AD patients who are refractory to traditional therapy.^2^, ^12^, ^13^ The present group has previously demonstrated such a finding.^14^

The authors evaluated the effect of MTX on the cutaneous expression of cytokines and chemokines that are involved in the inflammatory response in adult AD subjects who received treatment with methotrexate for a total of 24 weeks.

## Methods

### Study design and subjects

A prospective single-institution study was performed at the Department of Dermatology. The study population included 12 adults (6: female; 6: male) with moderate to severe AD (according to the Hanifin & Rajka criteria). The authors evaluated the use of MTX for a total of 24 weeks.

Eligibility criteria included subjects who were at least 18 years of age, with moderate/severe AD based on severity scores: EASI (Eczema Area and Severity Index) ≥7.1, and SCORAD (SCOring Atopic Dermatitis) ≥ 25. Women of childbearing age were required to be using effective birth control and were confirmed to not be pregnant with a negative blood pregnancy test prior to the start of treatment. Exclusion criteria included a subject with any disease or condition that contraindicated the use of MTX (e.g., pancytopenia), HIV infection or other immunosuppressive condition, phototherapy, systemic corticosteroid use, cyclosporine use, and azathioprine or immunobiological therapy within the 12 weeks prior to the start of MTX treatment, as detailed elsewhere.^14^, ^15^

The mean initial EASI of AD patients was 28.9 ± 9.9 and the mean initial SCORAD was 57.2 ± 7.8.^14^ The initial Methotrexate (oral test dose) was 7.5 mg in week 1, which was increased to 15 mg weekly. Oral folic acid was administered once a week (5 mg) 48–72 hours after MTX administration. During treatment, subjects continued standard of care AD topical medications, including topical corticosteroids, betamethasone valerate 0.1 % cream, hydrocortisone acetate 1.0% cream, calcineurin inhibitors, and emollients.

Subjects were biopsied in two different sites (lesional skin and skin with no clinical lesion). For the biopsy of the lesional skin, a non-photo exposed body region was selected, especially on the lower back. The investigation was performed on pre-treatment and after 24 weeks of MTX use. Skin samples of 10 non-atopic controls, matched by gender and age, performed in a similar anatomical site of AD patients, were used to standardize analyses.

All subjects read the informed consent and agreed to participate in the study. The study was approved by the local ethics committee (CAPPESq 13368); ClinicalTrial.gov identifier NCT03327116.

### Mean epidermal thickness and immunohistochemistry

Four-millimeter skin samples were obtained and embedded in paraffin for evaluation with immunohistochemistry. DAB (3.3′ diamibenzidine, D5637, Sigma, St. Louis, MO, USA) was used as a chromogen solution, as described previously.^15^, ^16^ After staining, the slides were scanned using Pannoramic Scan – 3Dhitech slide scanner (3DHistech Ltd., Budapest, Hungary).

The photos were assessed for immunohistochemical markings and mean epidermal thickness using the Image-Pro Plus program, version 4.5.0.29 (Media Cybernetics Inc., Bethesda, Maryland, USA).^17^

Mean epidermal thickness was calculated by drawing 2 lines: one in the upper part of the epidermis and one in the lower part of the epidermis, following the basement membrane. The average distance between these 2 lines was obtained with the average distance function of the Image-Pro-Plus program. This function measures the point-to-point distance between the 2 lines, at each angle variation, generates an average value, and calculates the mean epidermal thickness.

The primary antibodies IL-31, IL-31RA, OSMR, TSLP, and Ki-67 are listed in Table 1.

**Table 1:** Antibodies that were used for immunohistochemistry

Antibody	Dilution	Code	Brand	Positive control
IL-31	1:6	MAB28241	R&D Systems	AD
IL-31RA	1:10	AF-2769	R&D Systems	AD
OSMR	1:50	10982-1-AP	Proteintech	AD
TSLP	1:200	sc-33791	Santa Cruz	Prostate
Ki-67	1:200	275R-15	Cell Marque	Amygdala

AD, atopic dermatitis.

### Real-time PCR

mRNA expression by Real-Time PCR was performed using skin specimens that were stored in RNAlater solution (Sigma, Steinheim, Germany) and frozen at −80 °C. Total RNA was extracted utilizing an RNAeasy Plus Mini Kit (Qiagen, Valencia, CA, USA). cDNA was synthesized using the ISCRIPT cDNA KIT (Bio-rad, Hercules, CA, USA).

Synthesis of sense and antisense primers for IL-4, IL-6, IL-10, IL-31, IL-31RA, TNF-α, IFN-γ, TARC, CCL-22 and internal controls GAPDH (Glyceraldehyde -3-Phosphate Dehydrogenase) was performed by Invitrogen (Carlsbad, CA).^15^

PCR amplification was conducted in an Applied Biosystems 7500 system using the primers and SYBR Green (Applied Biosystems, Carlsbad, CA, USA) fluorescence detection reagents. The cycling protocol consisted of 10 min at 95 °C, followed by 40 cycles of 15 s at 95 °C and 60 s at 60 °C.

Sequence Detection System (SDS) software (Applied Biosystems) was used to analyze the results and were normalized.^18^

### Statistical analysis

Comparison between related samples (same subject, pre-treatment vs. post-treatment) was performed using the non-parametric Wilcoxon test. For unrelated samples, the Mann-Whitney was used. An alpha value was 0.05 throughout.

## Results

### Mean epidermal thickness

The average epidermal thickness in the lesional skin of AD subjects before treatment was 127.6 ± 194.3 μm. The average epidermal thickness in the lesional skin in AD subjects after MTX treatment was 100.3 ± 80.7 μm. Average epidermal thickness was significantly smaller in post-treatment lesional skin (mean difference = 31.3 μm, 95% CI 15.0–92.7; p = 0.021).

### Immunohistochemistry

**Figure 1:**
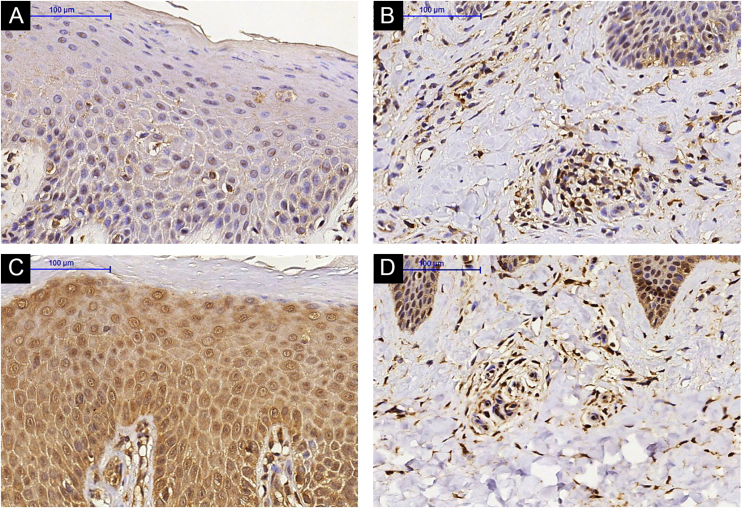
IL-31RA immunohistochemistry in lesional skin of an Atopic Dermatitis (AD) subject, epidermis (A) and superficial dermis (B). (A and B) Pre-Methotrexate (MTX) treatment; lesional skin of an AD subject, epidermis (C) and superficial dermis (D). (C and D) Post-MTX treatment (200× magnification)

**Figure 2:**
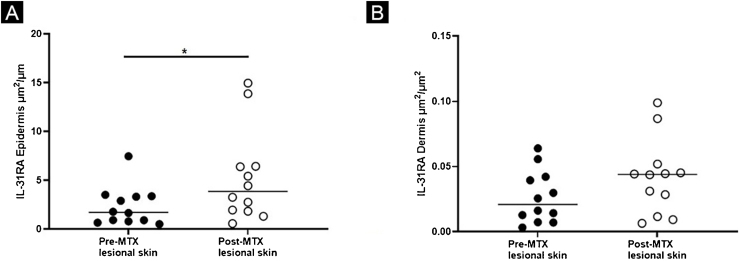
Expression of IL-31RA, comparing lesional skin before and after MTX treatment demonstrating a statistically significant change in the expression in the epidermis (A), with no significant difference in the superficial dermis (B). Lines represent cytokine medians in the specimens (*p < 0.05)

**Figure 3:**
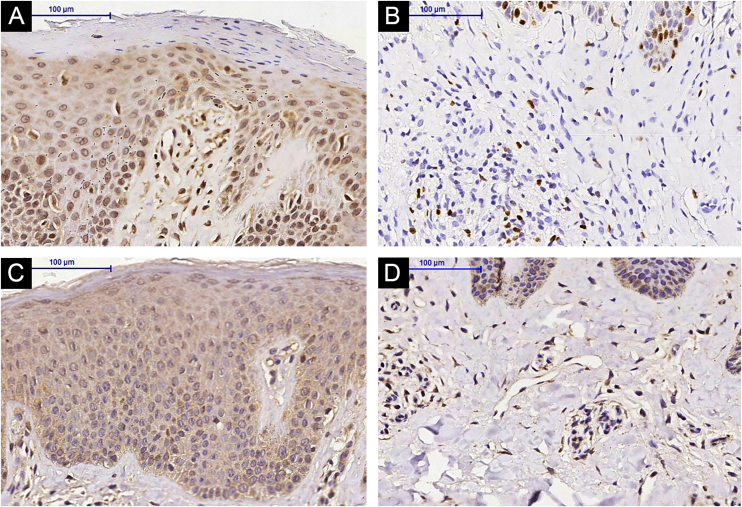
IL-31 immunohistochemistry in lesional skin of an Atopic Dermatitis (AD) subject, epidermis (A) and superficial dermis (B), (A and B) Pre-Methotrexate (MTX) treatment; lesional skin of an AD subject, epidermis (C) and superficial dermis (D), (C and D) Post-MTX treatment (200× magnification)

**Figure 4:**
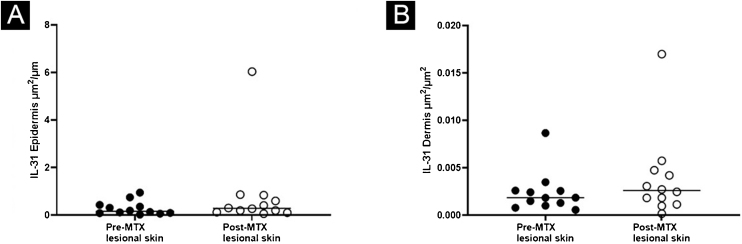
Expression of IL-31, comparing lesional skin before and after MTX treatment demonstrating a statistically significant change in the expression in the epidermis (A), with no significant difference in the superficial dermis (B). Lines represent cytokine medians in the specimens (*p < 0.05)

There was increased expression of IL-31RA in the epidermal skin (p = 0.016) from AD subjects post-MTX treatment and no significant difference in the superficial dermis (p = 0.151) (Figure 1, Figure 2). Cutaneous expression of the other markers did not reveal significant changes (Figure 3, Figure 4: IL-31; OSMR, TSLP, and Ki-67).

### Real-time PCR

**Figure 5:**
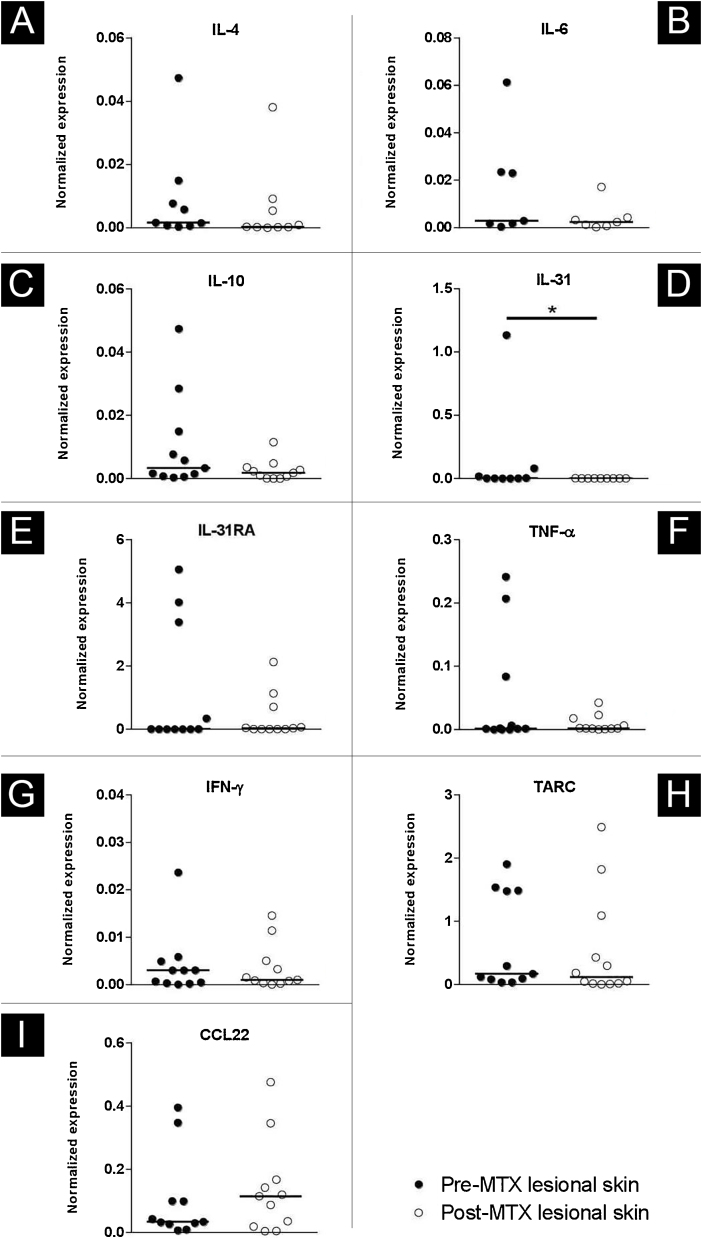
Expression profile of IL-4 (A), IL-6 (B), IL-10 (C), IL-31(D), IL-31RA (E), TNF-α (F), IFN-γ (G), TARC (H) and CCL-22 (I), comparing the lesional skin of an Atopic Dermatitis (AD) subject Pre-Methotrexate (MTX) treatment and post-MTX treatment by Real Time-PCR. There was a statistically significant decrease in IL-31 expression in lesional skin after treatment. Normalized expression was calculated as previously described.^18^ Lines represent cytokine medians in the skin specimens (*p < 0.05)

There was a decrease in the genic expression of IL-31 (p = 0.019) in lesional skin from AD subjects 24 weeks after MTX therapy. Cutaneous expression of the other evaluated markers (IL-4, IL-6, IL-10, IL-31RA, TNF-α, IFN-γ, TARC, and CCL-22) were not significant results (Fig. 5).

## Discussion

AD is a chronic and pruritic skin disease that is commonly observed in children; however, it may persist into or occur in adulthood. Quality of life is significantly impacted.^19^, ^20^ First-line treatment includes topical corticosteroids and emollient use. In moderate to severe cases that are refractory to conventional treatment, second-line medications, such as methotrexate, may be used.^12^, ^13^

There are few studies that evaluate the effect of MTX on cutaneous inflammatory mediators involved in AD patients. Schram et al. randomized adults with AD to receive MTX or azathioprine for 12 weeks and found a reduction in serum levels of TARC (Thymus and Activation-Regulated Chemokine) in both groups.^21^ Roekevisch et al. studied the effects of MTX and azathioprine in adult AD patients and observed significant reduction in TARC, CTACK (Cutaneous T-Cell-Attracting Chemokine or Chemokine [C-C motif] ligand 27 – CCL27), IL-13, and VEGF (*Vascular Endothelial Growth Factor*).^22^

The authors previously demonstrated the effectiveness of MTX in moderate/severe AD, finding a reduction in pruritus and symptom severity scores.^14^ Here, the authors show that cutaneous changes can be detected in AD subjects who are receiving MTX. The authors identified a reduction in mean epidermal thickness of lesional AD skin, an increase in IL-31RA expression by immunohistochemistry, and a decrease in gene expression of IL-31 by RT-PCR.

Reduction in mean epidermal thickness of lesional AD skin has not previously been shown and can be associated with improvement of signs and symptoms (pruritus) that are observed in AD patients.^14^ Similar results have been reported with other treatment modalities, including topical Phototherapy (PUVA) excimer laser, topical crisaborole, and dupilumab.^23^, ^24^, ^25^, ^26^

The reduction in pruritus observed after MTX treatment may be influenced, in part, by the reduction in IL-31 gene expression. IL-31 is involved in immunologic polarization towards the Th2 profile in the acute phase of AD, as well as in the pruritus of AD patients.^10^, ^27^, ^28^ Signaling occurs through a heterodimeric receptor. The IL-31RA subunit seems to play a more important role in the pathogenesis of AD than the OSMR subunit. 11, 29, 30

Identification of increased expression of IL-31RA in the epidermis, as seen with the immunohistochemistry, suggests a compensatory mechanism of IL-31RA, given the reduction in IL-31 gene expression. Upregulation of IL-31RA may occur after some stimuli: Mike et al. demonstrated IL-31RA upregulation after IL-4 stimulation in murine bone marrow-derived dendritic cells.^31^ Moreover, Edukulla et al. observed in mice that IL-4 and IL-13 could increase IL-31RA expression in macrophages of peritoneal and bone marrow.^32^

Potential limitations of the present study include a small sample size and limited length of follow-up; it is possible that a large-scale study with longer follow-ups would allow us to track changes in the cutaneous expression of inflammatory markers in AD in patients on MTX. New targeted therapies on the run and precision medicine may enhance the treatment of AD,^13^ alleviating the chronic course and the burden of this pruritic recalcitrant disease. Once access to such targeted therapies is still limited, identifying the immunological impact of the available therapies such as MTX for AD is justified.

## Conclusion

MTX in subjects with moderate/severe AD reduced epidermal hyperplasia and altered the expression of inflammatory cytokines and receptors that are related to pruritus, including IL-31 and IL-31RA.

## Financial support

This study was supported by Fundo de Apoio à Dermatologia de São Paulo (23‒2015).

## Authors’ contributions

Luciana Paula Samorano: Critical literature review; data collection, analysis, and interpretation; intellectual participation in propaedeutic and/or therapeutic management of studied cases; preparation and writing of the manuscript; statistical analysis; study conception and planning.

Kelly Cristina Gomes Manfrere: Data collection, analysis, and interpretation; effective participation in research orientation.

Naiura Vieira Pereira: Data collection, analysis, and interpretation; effective participation in research orientation.

Roberto Takaoka: Data collection, analysis, and interpretation; effective participation in research orientation.

Neusa Yuriko Sakai Valente: Data collection, analysis, and interpretation; effective participation in research orientation.

Mirian Nacagami Sotto: Data collection, analysis, and interpretation; effective participation in research orientation.

Luiz Fernando Ferraz Silva: Intellectual participation in propaedeutic and/or therapeutic management of studied cases; study conception and planning; effective participation in research orientation.

Maria Notomi Sato: Data collection, analysis, and interpretation; effective participation in research orientation.

Valeria Aoki: Approval of the final version of the manuscript; critical literature review; effective participation in research orientation; intellectual participation in propaedeutic and/or therapeutic management of studied cases; manuscript critical review; study conception and planning.

## Conflicts of interest

None declared.
